# From challenge to competence: the role of learning engagement in mediating stress and performance among clinical medical students in English-medium dental education

**DOI:** 10.3389/fmed.2025.1675855

**Published:** 2025-09-10

**Authors:** Runheng Liu, Jiahui Lin, Xiaoyan Chen, Yuhan Hou, Guanqi Liu, Yan Wang

**Affiliations:** Hospital of Stomatology, Guanghua School of Stomatology, Sun Yat-sen University, Guangzhou, China

**Keywords:** English-medium instruction, dental education, medical students, engagement, stress, academic performance

## Abstract

**Background:**

The adoption of English-medium instruction (EMI) in medical education has sparked ongoing debate, particularly in non-native English-speaking contexts. While EMI is increasingly applied to clinical medical programs, its effectiveness within dental education remains underexplored, especially with regard to changes over the duration of a course. This study aimed to examine the dynamic impact of instructional language (EMI vs. CMI) on medical students’ perceived academic stress, self-regulated learning engagement (SRLE) and academic performance in a dental course.

**Materials and methods:**

A quasi-experimental design was employed with 123 undergraduate medical students enrolled in a stomatology course. Participants were allocated to an EMI group (*n* = 41) or a Chinese-medium instruction (CMI) group (*n* = 82). Perceived stress and SRLE were measured at two time points: 1 month after course initiation (T1) and upon course completion (T2). Academic performance was evaluated via final examination and structured review writing tasks. Statistical analyses included between- and within-group comparisons, correlation analysis, hierarchical regression, and mediation modeling.

**Results:**

At T1, EMI students reported significantly higher stress and SRLE levels compared to CMI peers. While stress decreased over time in both groups, EMI students maintained consistently high engagement. T2 engagement significantly predicted review writing performance, fully mediating the effect of instructional language. Notably, the EMI group achieved higher review scores, though no significant difference was found in final exam performance. Correlation patterns diverged over time, with stress positively associated with engagement only in the EMI group at T2.

**Conclusion:**

Despite elevated stress levels, EMI students demonstrated superior learning engagement and higher-order academic performance. These findings suggest that EMI, when supported by adaptive engagement, may effectively foster interdisciplinary competence in dental education. Tailored pedagogical strategies are warranted to optimize stress adaptation and enhance learning outcomes in EMI environments.

## Introduction

With the increasing international exchange and cooperation in the medical field, English has become the dominant global language in specialized disciplines, including oral medicine (1). Consequently, to cultivate healthcare professionals capable of navigating this globalized environment, many non-native English-speaking countries have increasingly adopted English as the Medium of Instruction (EMI) in their higher education health science programs (2). In China, Chinese-Medium Instruction (CMI) has long been the standard approach in medical education, serving as the principal means of ensuring accessibility and comprehension for domestic students. However, in recent years, the national push toward internationalization and the desire to improve global competitiveness have led an increasing number of Chinese institutions to adopt EMI, aiming to enhance students’ English proficiency, facilitate access to international medical literature, and expand their career prospects (2, 3).

While the benefits of EMI are widely recognized, the transition is not without controversy. Studies indicate that EMI may pose barriers to effective teacher-student interaction and impede the comprehension of complex medical concepts, particularly for students who are less proficient in English (4). Research by Xu et al. found that students enrolled in EMI reported significantly higher anxiety and stress, which correlated with lower satisfaction and perceived learning achievement (5). However, educational psychology also highlights the potential for moderate levels of stress to act as “productive stress”—stimulating physiological and cognitive arousal that can enhance focus, motivation, and learning outcomes when managed effectively (6, 7).

The Challenge-Hindrance Stress Model provides a useful framework for understanding these dynamics. According to this model, “challenge stressors” such as demanding coursework or language barriers can be perceived as opportunities for growth and development, promoting adaptive coping strategies and engagement, whereas “hindrance stressors” are seen as obstacles that sap motivation and undermine performance (8). In the context of EMI, language-related demands may serve as challenge stressors for some students, encouraging them to adopt self-regulated learning strategies that ultimately improve academic outcomes. Conversely, for students who perceive these demands as insurmountable hindrances, stress may become maladaptive and detrimental to learning.

Despite growing attention to the role of stress in EMI settings, relatively little is known about how stress interacts with learning engagement and academic performance, particularly in non-native English contexts or within specialized fields such as dental education. Most existing studies have relied on cross-sectional designs and have not captured the dynamic processes by which students adapt over time (9). Furthermore, there remains a gap in understanding how adaptive engagement might mediate the relationship between stress and performance in EMI environments.

Stomatology is an essential part of clinical medicine. Growing evidence has revealed significant correlations between oral diseases and systemic conditions, such as the relationships of periodontitis with diabetes, cardiovascular diseases, and Alzheimer’s disease (10–12). Therefore, incorporating stomatology courses into clinical medicine curricula can enhance students’ comprehensive clinical competencies, foster a holistic medical perspective, and broaden their medical horizons and thinking patterns (13, 14). However, as a first-level discipline parallel to clinical medicine, stomatology encompasses extensive content with abstract theoretical knowledge, potentially posing substantial learning challenges for non-native English speakers in EMI settings (9, 15). Although the EMI approach has been widely adopted in clinical medical education, its effectiveness in specialized stomatology education remains unexplored, particularly regarding medical students’ learning experiences, academic performance, and instructional outcomes in English-taught stomatology courses.

This study helps to address these gaps by tracking cohorts in EMI and CMI models across sequential stages of a stomatology curriculum (T1 = 1 month after course commencement, T2 = at the end of the course). Based on the Perceived Stress Scale-10 (PSS-10) and Self-Regulated Learning Engagement (SRLE), we devised questionnaires to assess students’ perceived stress and learning engagement. At the T2 phase, the students’ academic cognition, interdisciplinary integration capacity, and critical thinking skills under different instructional modes is further analyzed, utilizing final examination scores and the review—writing performance. Through integrated analysis, this study examines: (1) the impact of stress on learning engagement in EMI contexts, and (2) the mediating role of adaptive engagement in shaping learning outcomes. These findings provide empirical evidence to inform the design and implementation of EMI curricula in medical education, with the goal of optimizing student adaptation and fostering globally competent healthcare professionals.

## Materials and methods

### Study design and participants

This quasi-experimental study was conducted among medical students enrolled in a stomatology course at Sun Yat-sen University. Participants were divided into two groups based on their enrollment: the English-medium instruction (EMI) group (n = 41) and the Chinese-medium instruction (CMI) group (n = 82). Students were free to choose whether to enroll in the EMI or CMI group based on their own preferences. All students followed the same curriculum, taught by faculty members with equivalent qualifications (each taught both EMI and CMI classes), differing only in the language of instruction. The study was conducted across one academic semester, with two data collection points: 1 month after course commencement (T1) and at the end of the course (T2). All participants provided written informed consent prior to participation. The study protocol was approved by the Ethics Committee of the Hospital of Stomatology, Sun Yat-sen University, and complied with the Declaration of Helsinki.

### Instructional context

The stomatology course was designed as an interdisciplinary module for clinical medicine students, covering dental anatomy, oral diseases, and oral-systemic health correlations. Course content, contact hours, assessments, and learning objectives were identical across both EMI and CMI groups. Instruction in the EMI group was entirely conducted in English, while the CMI group received instruction in Mandarin Chinese.

### Measures and instruments

The six perceived stress items were adapted from the Perceived Stress Scale-10 (PSS-10) (16), modified to suit the academic context of the stomatology course. The six learning engagement items were developed based on the self-regulated learning (SRL) framework, drawing on elements from existing SRL engagement questionnaires (17, 18), and refined through expert review and pilot testing. Each item was rated on a 0–10 numeric scale, with higher scores indicating greater perceived stress or engagement. This approach is analogous to a numerical rating scale (NRS) commonly used for subjective assessments (Table 1).

**Table 1:** Items used to assess perceived academic stress and learning engagement.

Domain	Item	Statement
Perceived academic stress	Q1	In the past month, how often have you felt confused or unable to grasp the knowledge points taught in this course?
	Q2	In the past month, how often have you felt it difficult to follow the course content or complete learning tasks?
	Q3	In the past month, how often have you felt overwhelmed by abstract or complex theoretical concepts in this course?
	Q4	In the past month, how often have you found it hard to balance other activities because of the time required for this course?
	Q5	In the past month, how often have you felt that the workload of this course was excessive or beyond your capacity to manage?
	Q6	In the past month, how often have you worried about your ability to perform well in assessments (e.g., exams, assignments) for this course?
Learning engagement	Q7	How much do you value the inclusion of practical courses (e.g., clinical observation) for enhancing your understanding of medical knowledge in this course?
	Q8	To what extent do you find the content of this course intrinsically interesting or enjoyable?
	Q9	How frequently do you preview learning materials (e.g., readings, lecture notes) before classes to prepare for upcoming topics?
	Q10	During lectures, how often do you actively focus on the instructor’s explanations and avoid distractions?
	Q11	After classes, how frequently do you use resource management strategies (e.g., consulting instructors, peer discussions, searching academic resources) to deepen your understanding of complex topics?
	Q12	To what extent has this course expanded your ability to think critically about interdisciplinary connections in medical practice?

### Academic performance metrics

Two forms of academic performance were evaluated: Review Writing Task: At T2, students completed a structured academic synthesis essay. Two independent, blinded raters assessed the submissions based on a standardized rubric measuring critical thinking, synthesis, and clarity (see Appendix 1 for details). To ensure the consistency of rating, we assessed inter-rater reliability using Cohen’s kappa statistic, which demonstrated excellent agreement between raters (Cohen’s kappa = 0.993). Final Examination: The final written examination consisted of objective questions assessing factual recall and clinical application based on the course content. All exam papers were graded by a single instructor to ensure consistency. The final examination was also scored on a 100-point scale.

### Statistical analyses

Descriptive statistics were calculated for demographic variables and all measured outcomes. Normality was assessed using the Shapiro–Wilk test. Depending on data distribution, independent samples t-tests or Mann–Whitney U tests were applied to compare EMI and CMI groups at each time point. Paired samples t-tests or Wilcoxon signed-rank tests were used to evaluate within-group changes over time.

Pearson correlation coefficients were calculated to explore relationships between stress, engagement, and academic scores within each group and time point. Fisher’s Z transformation was used to compare correlation coefficients across groups.

To examine the predictive effects of stress and engagement on academic performance, hierarchical multiple linear regression analyses were conducted. Instructional language was entered as a covariate in follow-up models. A mediation analysis using the causal steps approach and regression-based path modeling was employed to test whether T2 engagement mediated the effect of instructional language on review writing performance.

All statistical analyses were performed using SPSS version 29.0 and Python 3.9. Statistical significance was set at *p* < 0.05.

## Results

### Baseline equivalence and sample characteristics

A total of 123 medical students enrolled in a stomatology course participated in the study, comprising 41 students in the EMI group and 82 in the CMI group. Baseline demographic analysis revealed no significant differences between groups in terms of gender distribution or attrition rates across the study period (Table 2), ensuring the comparability of the two instructional groups.

**Table 2:** Baseline characteristics and follow-up stability of participants in EMI and CMI groups.

Group	T1		T2		Dropout rate
	Male	Female	Male	Female	
EMI (*n* = 41)	16	25	16	25	0%
CMI (*n* = 82)	33	49	33	49	0%
*p* value	1.000		1.000		

### Instrument reliability and structural validity

At T1, the questionnaire demonstrated excellent internal consistency, with a Cronbach’s alpha of 0.881 and a standardized alpha of 0.885, both exceeding the conventional threshold of 0.70 (Table 3). The Kaiser-Meyer-Olkin (KMO) measure yielded a value of 0.898, indicating sampling adequacy, while Bartlett’s test of sphericity was statistically significant (χ^2^ = 520.511, df = 66, *p* < 0.001), affirming the scale’s structural validity (Table 4). These findings confirm the robustness and psychometric appropriateness of the instrument for subsequent empirical analyses.

**Table 3:** Internal Consistency Reliability of the Questionnaire at T1.

Cronbach’s α	Standardized Cronbach’s *α*	Number of items	Sample size
0.881	0.885	12	123

**Table 4:** KMO and Bartlett’s test of sphericity for the questionnaire at T1.

KMO value		0.898
Bartlett’s test of sphericity	Approx. Chi-Square	520.511
	df	66
	*p* value	<0.001^***^

*** Indicates *p* < 0.001.

### Group differences in stress and engagement over time

Comparisons across time points revealed dynamic patterns in perceived stress and self-regulated learning engagement (SRLE) across groups and time points (Table 5). At baseline (T1), the EMI group reported significantly higher stress levels compared to the CMI group (50.49 ± 2.31 vs. 31.45 ± 5.26, *p* < 0.001), which persisted-albeit reduced-at T2 (34.66 ± 3.90 vs. 30.96 ± 4.56, *p* < 0.05).

**Table 5:** Comparison of stress and engagement scores between EMI and CMI groups at T1 and T2 (Mean ± SD).

Group	T1 stress	T2 stress	T1 engage	T2 engage
CMI (*N* = 82)	31.45 ± 5.26	30.96 ± 4.56	26.22 ± 2.94	28.68 ± 4.65
EMI (*N* = 41)	50.49 ± 2.31	34.66 ± 3.9	40.27 ± 4.72	40.71 ± 2.62

**Figure 1:**
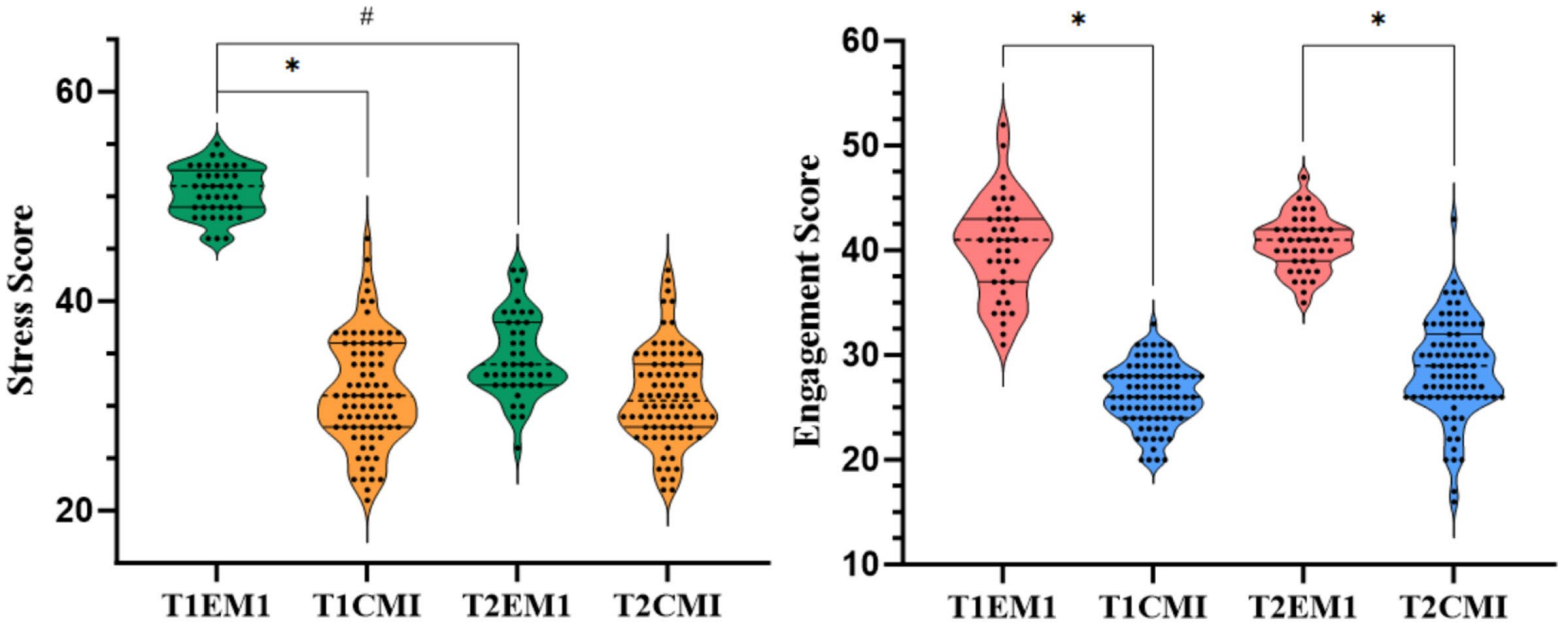
Stress and engagement scores at T1 and T2 across CMI and EMI groups. Boxplot comparing stress scores between CMI and EMI students at two time points: T1 and T2. * indicates between-group differences at the same time point (*p* < 0.05); # indicates within-group differences across time points (*p* < 0.05).

In contrast, engagement scores were consistently higher in the EMI group at both T1 and T2 (T1: 40.27 ± 4.72 vs. 26.22 ± 2.94; T2: 40.71 ± 2.62 vs. 28.68 ± 4.65; both *p* < 0.001). A significant within-group increase in SRLE from T1 to T2 was noted in the CMI group (*p* < 0.05), whereas EMI students maintained a high but stable engagement level. These data indicate that despite initially elevated stress levels, EMI students exhibited more sustained and intensive learning behaviors over time (Figure 1).

### Correlational patterns between stress and engagement

**Figure 2:**
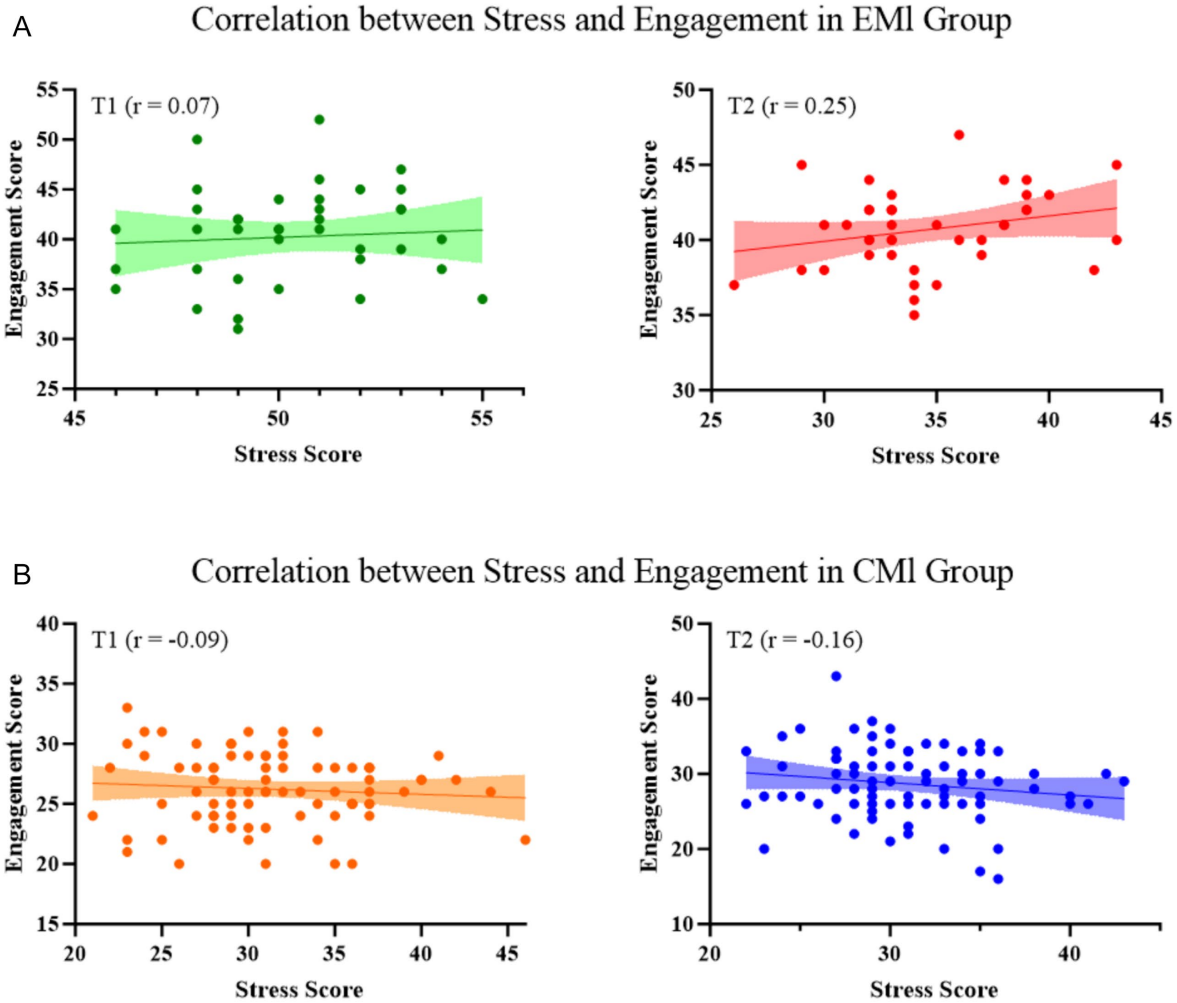
Correlation between stress and engagement at two time points (T1 and T2) in EMI and CMI groups. **(A)** Scatter plots with regression lines showing the relationship between stress and engagement in the EMI group at T1 (green) and T2 (red). **(B)** Corresponding plots for the CMI group at T1 (orange) and T2 (blue).

**Figure 3:**
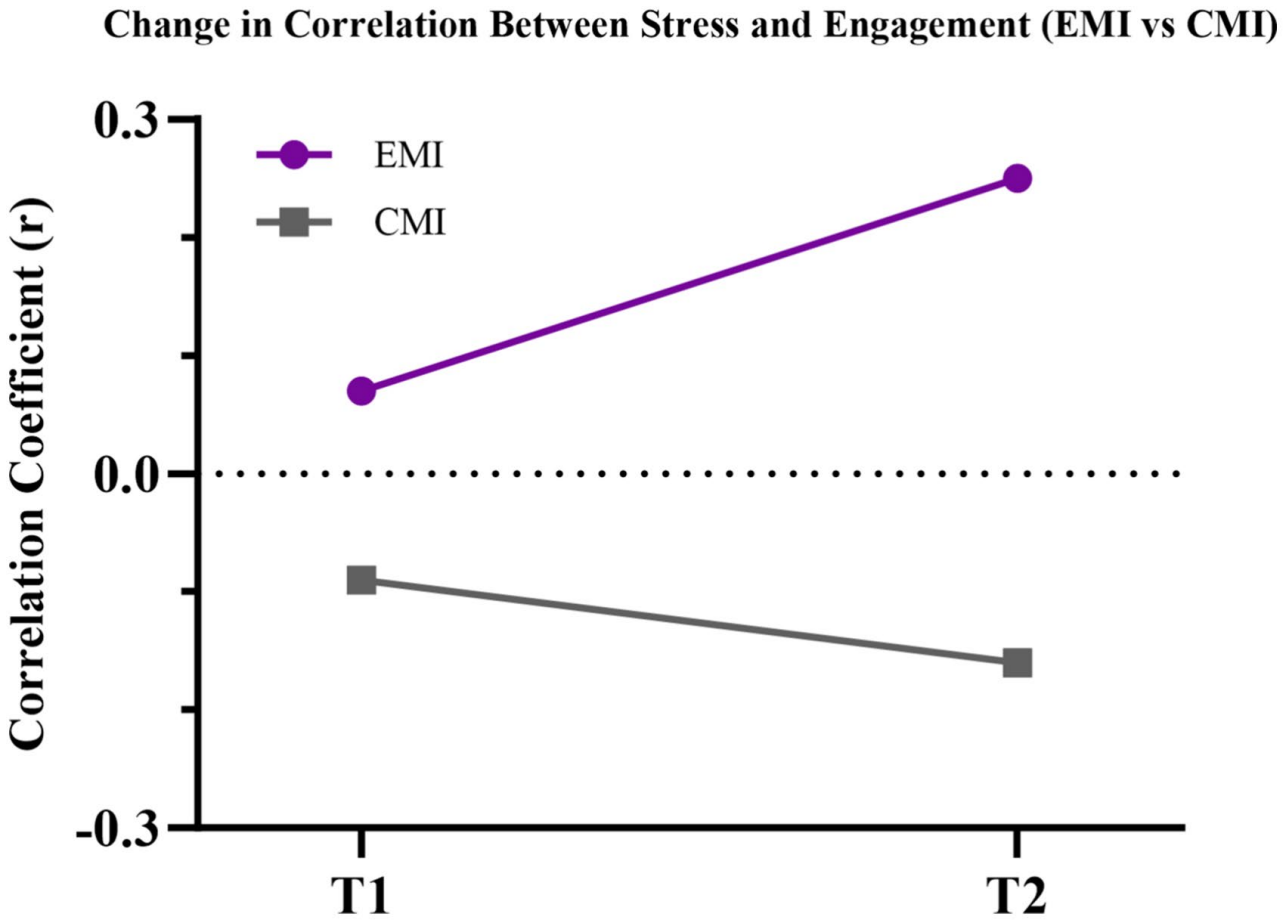
Line graph depicting the change in Pearson correlation coefficients over time within each group.

To elucidate the interaction between stress and engagement, Pearson’s correlations were calculated separately by instructional group and time point. At T1, no significant associations were observed (EMI: r = 0.07; CMI: r = −0.09). However, by T2, a positive correlation emerged in the EMI group (r = 0.25), whereas a negative trend persisted in the CMI group (r = −0.16). Fisher’s Z transformation confirmed a significant between-group difference in correlation strength at T2 (z = 2.13, *p* = 0.033), but not at T1 (Figures 2, 3; Table 6). These findings suggest divergent psychological adaptations to academic stress in EMI vs. CMI contexts.

**Table 6:** Fisher’s Z test results comparing the strength of correlations between groups at T1 and T2.

Time point	Z score	*p* value
T1	0.81	0.418
T2	2.13	0.033^*^

* Indicates *p* < 0.05.

### Academic performance comparisons

**Figure 4:**
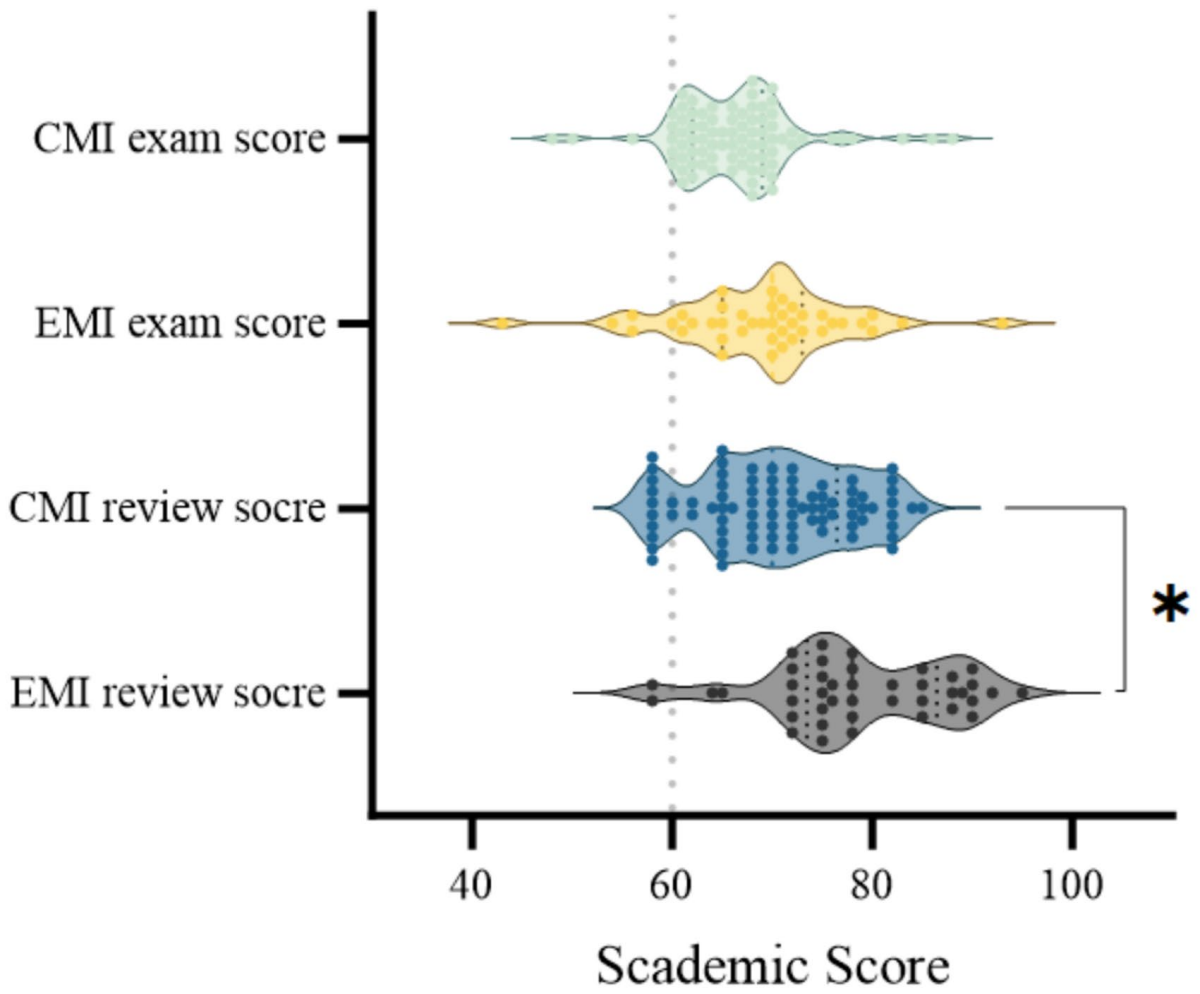
Violin plots showing the distribution of academic scores in the CMI and EMI groups. Each violin represents the density and spread of individual scores for the final exam and review writing. * indicates *p* < 0.05.

Comparisons of academic outcomes revealed that the EMI group significantly outperformed the CMI group in review writing scores (78.68 ± 8.86 vs. 70.50 ± 7.61, *p* < 0.0001, Cohen’s d = 1.01). According to conventional benchmarks, this represents a large effect size, indicating a substantial practical difference between groups in higher-order academic skills. For the comparison of final exam scores between the EMI and CMI groups, the difference was not statistically significant (68.91 ± 8.75 vs. 66.26 ± 6.21, *p* = 0.055, Cohen’s d = 0.37) (Table 7; Figure 4). This suggests that the EMI instruction model may better foster higher-order competencies such as synthesis, critical thinking, and written expression, while not necessarily conferring advantages in factual recall-based examinations. This suggests that the EMI instruction model may better foster higher-order competencies such as synthesis, critical thinking, and written expression, while not necessarily conferring advantages in factual recall-based examinations.

**Table 7:** Comparison of academic outcomes between CMI (Chinese-medium instruction) and EMI (English-medium instruction) groups (Mean ± SD).

Group	CMI (*N* = 82)	EMI (*N* = 41)	*p* value
Final exam	66.26 ± 6.21	68.91 ± 8.75	0.055
Review writing	70.50 ± 7.61	78.68 ± 8.86	<0.0001^*^

* Indicates *p* < 0.05.

### Predictors of academic writing performance

Correlation analysis revealed that T2 engagement was the strongest predictor of review writing performance (r = 0.465, *p* < 0.001), followed by T1 engagement (r = 0.396) and T1 stress (r = 0.387). T2 stress showed a weaker and non-significant association (r = 0.162, *p* = 0.073) (Table 8).

**Table 8:** Pearson correlation between stress, engagement, and review scores (*n* = 123).

Variable	Pearson *r*	*p* value
T1 engagement	0.396	<0.001^***^
T2 engagement	0.465	<0.001^***^
T1 stress	0.387	<0.001^***^
T2 stress	0.162	0.0734

*** Indicates *p* < 0.001.

A multiple linear regression model confirmed that only T2 engagement significantly predicted review writing scores (*β* = 0.438, *p* = 0.008), accounting for 22.7% of the variance (Table 9). Adding instructional language as a covariate did not improve model fit nor did it emerge as a significant predictor, suggesting that its effect was fully mediated by post-course engagement (*β* = 1.273, *p* = 0.838) (Table 10).

**Table 9:** Multiple linear regression predicting review writing scores from stress and engagement measures (*n* = 123).

Predictor	β coefficient	*p* value
T2 engagement	0.438	0.008^**^
T1 engagement	0.098	0.567
T1 stress	0.083	0.631
T2 stress	−0.035	0.882

R^2^ = 0.227; Adjusted R^2^ = 0.201; Model significance: F(4, 118) = 8.66, ^**^p < 0.001.

**Table 10:** Multiple linear regression including instructional language (*n* = 123).

Predictor	β coefficient	*p* value
T2 engagement	0.423	0.020^**^
T1 engagement	0.077	0.702
T1 stress	0.040	0.884
T2 stress	−0.003	0.991
Instructional language (EMI)	1.273	0.838

R^2^ = 0.229; Adjusted R^2^ = 0.196; Model significance: F(5, 117) = 6.96, ^**^p < 0.001.

### Mediation effect of engagement on EMI outcomes

**Figure 5:**
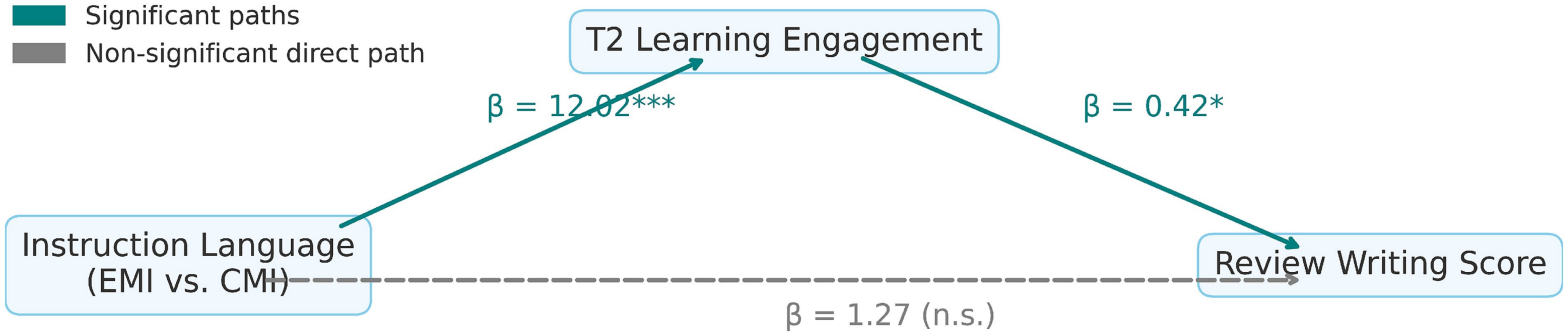
Final mediation model linking instructional language to review writing performance via T2 learning engagement.

Mediation analysis demonstrated a full mediating effect of T2 engagement on the relationship between instructional language and review writing performance. EMI significantly predicted greater post-course engagement (β = 12.02, *p* < 0.001), which in turn significantly enhanced review scores (β = 0.42, *p* = 0.020). The direct effect of EMI on review performance became non-significant when controlling for engagement (β = 1.27, *p* = 0.838), confirming full mediation (Figure 5; Table 11).

**Table 11:** Mediation path regression results.

Path	β coefficient	*p* value
Instruction language → Review (total effect)	8.18	<0.0001^***^
Instruction language → T2 engagement	12.02	<0.0001^***^
T2 engagement → Review (with Class controlled)	0.42	0.02^*^
Instruction language → Review (direct effect)	1.27	0.838

* Indicates *p* < 0.05, and *** Indicates *p* < 0.001.

## Discussion

### Principal findings and their implications

This prospective study investigated the dynamic effects of English-Medium Instruction (EMI) versus Chinese-Medium Instruction (CMI) on medical students’ perceived stress, learning engagement, and academic performance within a stomatology course. Key findings demonstrate that while EMI students experienced significantly higher academic stress at the beginning of the course, they also exhibited consistently elevated levels of self-regulated learning engagement (SRLE). Notably, this enhanced engagement mediated the positive effect of EMI on higher-order academic performance, as reflected in superior review writing scores, despite no significant difference in final exam outcomes between groups. These findings reveal a paradox wherein heightened stress often viewed as detrimental may serve as a catalyst for adaptive learning behaviors under appropriate pedagogical contexts. However, given the quasi-experimental nature of our study, these results should be interpreted as associations rather than definitive causal effects.

### Productive stress and adaptive engagement in EMI contexts

Our results align with theories in educational psychology suggesting that moderate stress can stimulate goal directed behaviors and cognitive arousal, thus enhancing learning performance (19). Students in the EMI group reported significantly greater stress and engagement at T1, potentially due to language-related challenges and cognitive demands associated with processing specialized medical content in a second language. These early difficulties appeared to trigger proactive coping strategies, including increased resource-seeking and autonomous learning behaviors, ultimately contributing to academic success.

This phenomenon may be understood within the framework of challenge–hindrance stress theory, wherein challenge stressors (e.g., complex content in EMI) are appraised as opportunities for growth, potentially promoting engagement and performance (8). The mediation analysis indicated that post-course engagement statistically mediated the association between instructional language and review scores, suggesting a possible mechanism by which EMI relates to academic outcomes. Nevertheless, due to the limitations of regression and mediation analyses in non-randomized designs, these findings should be viewed as indicative of possible pathways rather than evidence of direct causality.

### Differential effects on academic outcomes

The divergence between review writing and exam scores provides insight into the cognitive dimensions fostered by EMI. The EMI group’s superior performance in review tasks, which require synthesis, critical thinking, and structured communication, suggests that EMI may preferentially enhance higher-order cognitive skills. In contrast, the lack of significant group differences in final exam scores, which emphasize factual recall, implies that EMI does not necessarily affect rote learning. For example, Olop et al. and Lim et al. (20, 21) found that students in EMI contexts reported greater use of deep learning strategies compared to surface-level memorization, which is consistent with our findings.

Furthermore, the stronger correlation between post-course engagement and review performance underscores the importance of sustained effort over time. These findings support pedagogical models that emphasize formative assessments and integrative tasks in EMI curricula, which may better capture students’ intellectual growth than traditional summative tests.

### Instructional design considerations for EMI implementation

The study’s findings carry practical implications for designing EMI programs in non-native English contexts. Although EMI may initially elevate student stress levels, the provision of adequate scaffolding, such as supplemental language support, task-based instruction, and adaptive feedback, can help students channel this stress into productive learning behaviors. Institutions should also invest in promoting SRLE through reflective tasks, peer collaboration, and metacognitive training.

Moreover, instructional strategies should acknowledge individual variability in coping mechanisms and linguistic readiness. For example, the voluntary nature of group assignment in this study raises questions about self-selection bias and intrinsic motivation among EMI participants. Future research could explore how learner characteristics, such as academic self-efficacy, language proficiency, and cultural attitudes, interact with EMI to shape learning trajectories.

These findings suggest that for medical schools considering the implementation of EMI, it is essential to provide comprehensive support systems to facilitate student adaptation and success. Practical strategies may include: (1) offering preparatory academic English language courses prior to or alongside EMI programs; (2) providing ongoing language support and bilingual learning resources; (3) establishing peer mentoring and study groups to foster collaborative learning; and (4) training faculty in EMI pedagogy and culturally responsive teaching methods. Such measures can help mitigate language-related challenges, reduce stress, and promote greater engagement and academic achievement among students in EMI environments.

### Limitations and directions for future research

Several limitations should be noted. First, stress and engagement were assessed via self-report questionnaires, which may introduce response bias or be influenced by social desirability. Second, individual differences such as English language proficiency, prior educational experiences, and cultural background were not measured in this study, but could have affected students’ stress perceptions, engagement levels, and academic performance. Future research should include more objective measures and control for these factors to better elucidate the mechanisms underlying student adaptation in EMI contexts. Although regression and mediation analyses were conducted to explore relationships among variables, the quasi-experimental design limits the ability to infer causality. Our findings should therefore be interpreted as reflecting associations rather than causal relationships. Future studies employing randomized controlled designs or longitudinal interventions are needed to establish causality.

## Conclusion

This study provides evidence that English-medium instruction (EMI), despite initially elevating students’ academic stress, fosters sustained learning engagement and enhances higher-order academic performance among clinical medical students in a dental education context. The findings suggest that EMI environments, when supported by adequate pedagogical structures, can promote productive coping and self-regulated learning behaviors. Engagement emerged as a critical mediating factor linking instructional language to academic writing outcomes, underscoring its central role in curriculum design. However, the statistical analyses used in this study suggest possible associations and mediating pathways rather than definitive causal effects. These insights support the strategic implementation of EMI in medical education, with careful attention to learners’ psychological adaptation and engagement dynamics.

## Data Availability

The datasets presented in this study can be found in online repositories. The names of the repository/repositories and accession number(s) can be found in the article/Supplementary material.
