# Supplementary material for: From challenge to competence: the role of learning engagement in mediating stress and performance among clinical medical students in English-medium dental education
Source: Front Med (Lausanne). 2025 Sep 10;12:1675855. doi: 10.3389/fmed.2025.1675855 (PMC12457135; doi:10.3389/fmed.2025.1675855)
Supplement: Supplementary file 1 [file Table_1.DOCX]

Appendix 1. Standardized Rubric for Review Writing Task (Total: 100 points)

| Criterion | Description | Points |
| --- | --- | --- |
| Understanding of Key Concepts | Demonstrates accurate and comprehensive understanding of core course content and concepts. | 25 |
| Critical Analysis and Synthesis | Critically analyzes information, integrates relevant sources, and synthesizes ideas. | 25 |
| Organization and Structure | Presents ideas logically with clear structure (introduction, body, conclusion, transitions). | 20 |
| Use of Evidence and Examples | Supports arguments with appropriate evidence, examples, and references. | 15 |
| Clarity and Academic Language | Expresses ideas clearly using appropriate academic language and terminology. | 10 |
| Mechanics (Grammar, Spelling, Format) | Demonstrates correct grammar, spelling, and follows required formatting guidelines. | 5 |
| Total | | 100 |

Appendix 2. Review Writing Scores

| Student ID | Teacher_A | Teacher_B | Average |
| --- | --- | --- | --- |
| 1 | 92 | 88 | 90 |
| 2 | 87 | 89 | 88 |
| 3 | 84 | 86 | 85 |
| 4 | 80 | 76 | 78 |
| 5 | 77 | 73 | 75 |
| 6 | 83 | 81 | 82 |
| 7 | 76 | 74 | 75 |
| 8 | 83 | 87 | 85 |
| 9 | 74 | 76 | 75 |
| 10 | 59 | 57 | 58 |
| 11 | 70 | 74 | 72 |
| 12 | 87 | 83 | 85 |
| 13 | 71 | 73 | 72 |
| 14 | 89 | 87 | 88 |
| 15 | 70 | 74 | 72 |
| 16 | 79 | 77 | 78 |
| 17 | 77 | 73 | 75 |
| 18 | 64 | 66 | 65 |
| 19 | 75 | 77 | 76 |
| 20 | 60 | 56 | 58 |
| 21 | 74 | 70 | 72 |
| 22 | 86 | 84 | 85 |
| 23 | 80 | 76 | 78 |
| 24 | 75 | 77 | 76 |
| 25 | 63 | 65 | 64 |
| 26 | 71 | 73 | 72 |
| 27 | 73 | 71 | 72 |
| 28 | 87 | 91 | 89 |
| 29 | 80 | 76 | 78 |
| 30 | 94 | 90 | 92 |
| 31 | 76 | 80 | 78 |
| 32 | 88 | 92 | 90 |
| 33 | 79 | 77 | 78 |
| 34 | 77 | 73 | 75 |
| 35 | 81 | 83 | 82 |
| 36 | 92 | 88 | 90 |
| 37 | 76 | 74 | 75 |
| 38 | 94 | 96 | 95 |
| 39 | 73 | 77 | 75 |
| 40 | 90 | 86 | 88 |
| 41 | 89 | 91 | 90 |
| 42 | 83 | 81 | 82 |
| 43 | 74 | 76 | 75 |
| 44 | 62 | 58 | 60 |
| 45 | 67 | 63 | 65 |
| 46 | 73 | 77 | 75 |
| 47 | 74 | 70 | 72 |
| 48 | 68 | 72 | 70 |
| 49 | 76 | 72 | 74 |
| 50 | 66 | 70 | 68 |
| 51 | 67 | 63 | 65 |
| 52 | 65 | 67 | 66 |
| 53 | 60 | 56 | 58 |
| 54 | 80 | 78 | 79 |
| 55 | 74 | 78 | 76 |
| 56 | 81 | 77 | 79 |
| 57 | 68 | 72 | 70 |
| 58 | 66 | 64 | 65 |
| 59 | 72 | 68 | 70 |
| 60 | 68 | 72 | 70 |
| 61 | 63 | 65 | 64 |
| 62 | 67 | 69 | 68 |
| 63 | 67 | 63 | 65 |
| 64 | 64 | 66 | 65 |
| 65 | 80 | 84 | 82 |
| 66 | 86 | 84 | 85 |
| 67 | 81 | 83 | 82 |
| 68 | 60 | 56 | 58 |
| 69 | 57 | 59 | 58 |
| 70 | 67 | 63 | 65 |
| 71 | 69 | 67 | 68 |
| 72 | 78 | 78 | 78 |
| 73 | 68 | 72 | 70 |
| 74 | 71 | 69 | 70 |
| 75 | 84 | 80 | 82 |
| 76 | 76 | 80 | 78 |
| 77 | 56 | 60 | 58 |
| 78 | 83 | 81 | 82 |
| 79 | 81 | 83 | 82 |
| 80 | 70 | 66 | 68 |
| 81 | 82 | 86 | 84 |
| 82 | 80 | 76 | 78 |
| 83 | 84 | 80 | 82 |
| 84 | 67 | 69 | 68 |
| 85 | 73 | 71 | 72 |
| 86 | 78 | 80 | 79 |
| 87 | 60 | 56 | 58 |
| 88 | 81 | 83 | 82 |
| 89 | 69 | 67 | 68 |
| 90 | 70 | 74 | 72 |
| 91 | 80 | 76 | 78 |
| 92 | 77 | 79 | 78 |
| 93 | 56 | 60 | 58 |
| 94 | 74 | 76 | 75 |
| 95 | 81 | 79 | 80 |
| 96 | 71 | 73 | 72 |
| 97 | 66 | 64 | 65 |
| 98 | 63 | 61 | 62 |
| 99 | 77 | 73 | 75 |
| 100 | 74 | 78 | 76 |
| 101 | 80 | 76 | 78 |
| 102 | 59 | 57 | 58 |
| 103 | 61 | 63 | 62 |
| 104 | 74 | 70 | 72 |
| 105 | 72 | 76 | 74 |
| 106 | 57 | 59 | 58 |
| 107 | 67 | 63 | 65 |
| 108 | 68 | 72 | 70 |
| 109 | 69 | 71 | 70 |
| 110 | 74 | 76 | 75 |
| 111 | 73 | 71 | 72 |
| 112 | 67 | 63 | 65 |
| 113 | 64 | 66 | 65 |
| 114 | 66 | 64 | 65 |
| 115 | 60 | 56 | 58 |
| 116 | 57 | 59 | 58 |
| 117 | 62 | 58 | 60 |
| 118 | 74 | 72 | 73 |
| 119 | 71 | 73 | 72 |
| 120 | 66 | 70 | 68 |
| 121 | 69 | 67 | 68 |
| 122 | 74 | 70 | 72 |
| 123 | 72 | 76 | 74 |
